# Downstaging Therapies for Unresectable Hepatocellular Carcinoma Prior to Hepatic Resection: A Systematic Review and Meta-Analysis

**DOI:** 10.3389/fonc.2021.740762

**Published:** 2021-11-19

**Authors:** Xinyu Chen, Lin Lai, Jiazhou Ye, Lequn Li

**Affiliations:** ^1^ Department of Hepatobiliary Surgery, Guangxi Medical University Cancer Hospital, Nanning, China; ^2^ Department of Medical Oncology, The First Affiliated Hospital of Guangxi Medical University, Nanning, China; ^3^ Department of Radiotherapy, Guangxi Medical University Cancer Hospital, Nanning, China

**Keywords:** hepatic resection (HR), downstaging, hepatocellular carcinoma, unresectable, meta-analysis

## Abstract

**Introduction:**

Hepatocellular carcinoma (HCC) is a high-grade malignant disease with unfavorable prognosis, and although surgical therapy is necessary, not all patients with HCC are suitable candidates for surgery. Downstaging as preoperative therapeutic strategy, which can convert unresectable HCC into resectable HCC, intends to increase the resection rate and improve prognosis.

**Methods:**

We searched multiple databases updated to December 30, 2020, for studies on transcatheter arterial chemoembolization (TACE), Yttrium 90 microsphere selective internal radiation (SIR)/transcatheter radioembolization (TARE), hepatic arterial infusion (HAI), and systemic treatment as downstaging treatment before resection for patients with unresectable HCC.

**Results:**

A total of 20 comparative and non-comparative studies were finally included in the meta-analysis. The pooled downstaging rate of hepatic resection (HR) was 14% [95% confidence interval (CI) 0.10–0.17] with significant heterogeneity (*I* ^2^ = 94.51%). The chemotherapy, combination, and non-cirrhosis groups exhibit higher rates of downstaging, but these differences were not significant. For comparative studies, the overall survival (OS) rates of resection after downstaging were far better than those inpatients who received locoregional therapy (LRT) or systemic treatment alone at 1 year (RR 1.87, 95% CI 1.48–2.38), 3 years (RR 5.56, 95% CI 2.55–12.10), and 5 years (RR 5.47, 95% CI 2.22–13.49). In addition, the pooled disease-free survival (DFS) rates in patients undergoing HR after successful downstaging were 78% (95% CI 0.62–0.93) at 1 year, 47% (95% CI 0.25–0.68) at 3 years, and 46% (95% CI 0.32–0.59) at 5 years. The pooled OS rates were 88% (95% CI 0.82–0.95) at 1 year, 64% (95% CI 0.59–0.69) at 3 years, and 42% (95% CI 0.29–0.54) at 5 years.

**Conclusions:**

Downstaging may serve as a screening tool to identify patients who might benefit from surgery. Resection after successful downstaging can improve prognosis.

## Introduction

Hepatocellular carcinoma (HCC) is a malignant disease that ranks sixth in morbidity and fourth in fatality among cancers globally (1). Hepatic resection (HR) and liver transplantation (LT) are the main curative-intent options, which offer 5-year survival rate exceeding 70% in patients with early HCC. However, the application of LT is limited by a shortage of donors. Thus, HR is currently a popular curative therapy. The indications for HR in treating HCC remain controversial. According to guidelines from the European Association for the Study of the Liver (EASL) (2), American Association for the Study of Liver Disease (AASLD) (3), and National Comprehensive Cancer Network (NCCN) (4), following the Barcelona Clinic Liver Cancer (BCLC) system (5), only patients with stage A are resectable. Unfortunately, based on the indication, most patients miss the time window for surgical therapy, leaving less than 30% of patients’ resectability at the time of diagnosis.

In this context, the indications for HR extend beyond the early stage of HCC in clinical practice (6–8). The Asian Pacific Association for the Study of the Liver (APASL) guidelines set wider indications for HR (9); some patients with BCLC stages B–C can be considered candidates for resection in terms of tumor burden and liver functional reserves. Moreover, comprehensive strategies are required to create opportunities for resection. Therapies that include locoregional therapies (LRTs) such as transcatheter arterial chemoembolization (TACE), Yttrium 90 microsphere selective internal radiation (SIR)/transcatheter radioembolization (TARE), hepatic arterial infusion (HAI) for patients with stage B, and systemic treatment such as molecular targeting and chemotherapy for patients with stage C aim at reducing the tumor load and tumor stage to convert unresectable HCC to resectable HCC, or to make it easier to remove the tumor radically; this approach is referred to as downstaging (10). Downstaging strategies are recommended for patients exceeding the Milan criteria considered for LT by the EASL, AASLD, and NCCN (2–4). However, since most studies on downstaging of HR have had small sample sizes, inconsistent inclusion criteria, great difference in results, and lack of prospective clinical trials with large samples, no consensus has been reached yet on HR. There are no systematic reviews or meta-analyses of downstaging prior to HR in patients with advanced unresectable HCC. Therefore, we systematically summarized studies on downstaging therapies for HCC, to synthesize the existing evidence regarding the efficacy of LRT or systemic therapies as downstaging strategies for patients with unresectable HCC who are potential candidates for resection.

## Materials and Methods

All methods were performed according to the Preferred Reporting Items for Systematic Reviews and Meta-Analyses (PRISMA) (11) and MOOSE (Meta-analysis of Observational Studies in Epidemiology) (12) reporting guidelines.

### Literature Screening and Search Strategy

We thoroughly searched all relevant studies updated to December 30, 2020, in the PubMed (https://www.ncbi.nlm.nih.gov/pubmed), Cochrane (https://www.cochranelibrary.com), Embase (https://www.embase.com), Web of Science (https://www.webofknowledge.com), VIP (http://www.cqvip.com), Wanfang (http://www.wanfangdata.com.cn), and CNKI (https://www.cnki.net) database. The search strategy was as follows: [(hepatocellular carcinoma) OR (hepatocellular cancer) OR (liver cancer) OR (hepatic neoplasm)] AND [(downstaging) OR (downstage) OR (down stage) OR (conversion therapy) OR (preoperative treatment) OR (preoperative treatments) OR (preoperative therapy) OR (preoperative therapies)] AND [(hepatic resection) OR (hepatectomy) OR (salvage)].

### Study Selection

Two authors independently conducted the literature search and initially selected relevant studies by reading titles and abstracts. Studies describing irrelevant subjects were excluded from the first step. Furthermore, the remaining studies were further screened by reading the full texts, and ineligible studies were discarded. Our inclusion criteria were as follows: (1) patients who were diagnosed as unresectable HCC, including extrahepatic disease or extensive local disease not amenable to definitive resection; (2) intervention included any treatment for HCC that reduced the tumor load and tumor stage (e.g., TACE, radiofrequency ablation, SIR/TARE, systemic therapy, or a combination of therapies); (3) studies eligible for our meta-analysis included prospective and retrospective comparative studies, cohort observational studies, and case series; and (4) outcomes evaluating rates of success for downstaging, overall survival (OS), disease-free survival (DFS) rate, and recurrence-free survival (RFS) rate. We excluded articles that (1) included less than five patients; (2) reported duplicate cohorts of patients, in which case we used the most updated cohort or the most recent publication; (3) were conference proceedings, letters, literature reviews, systematic reviews, case reports, comments, animal experiments, or unpublished studies with no full-text availability; (4) applied no restrictions to the language of articles; (5) reported downstaging used in LT, unless the studies also included a resection group, the data of which could be used to evaluate the downstaging success rate or survival rate could be extracted separately.

### Data Extraction

For each study, data were extracted in duplicate using standardized forms. We extracted the following variables from each study: study characteristics (first author’s last name, publication year, study design, and sample size), downstaging treatment modality, downstaging rate, OS rate, and DFS rate. The outcomes we extracted from each study were as follows: (1) the success rate of downstaging to remove tumor completely and (2) the long-term survival for HR with downstaging.

### Quality Assessment

The quality of randomized controlled trials (RCTs) and non-randomized controlled trials (nRCTs) were assessed using the Cochrane bias assessment tool (13) and methodological index for non-randomized studies (MINORS) (14), respectively. The observational study quality was based on the modified Newcastle-Ottawa scale (NOS) (15). The Institute of Health Economics Quality Appraisal (IHEQA) Checklist (16) was used to assess methodological quality for case series studies without a control group.

### Statistical Analysis

For comparative studies, we calculated the relative risks (RRs) and 95% confidence intervals (CIs) employing a binomial distribution. For noncomparative studies, we calculated the event rates of outcomes, and we estimated 95% CIs using Jeffreys method. A heterogeneity test for homogeneity of effect size was also given. Heterogeneity was assessed by *I* ^2^ statistics and the *p*-value of the Chi-square test (17). A random-effects model was used to merge the data and estimate effect-size indicators according to the Cochrane Handbook for Systematic Reviews of Interventions version 6.2 (18). Statistical analysis was conducted using Stata 15.0 software (Stata Corporation, College Station, TX, USA), and a *p*-value <0.05 was considered statistically significant for the effect size of each included study.

## Results

### Study Characteristics and Quality Assessment

**Figure 1:**
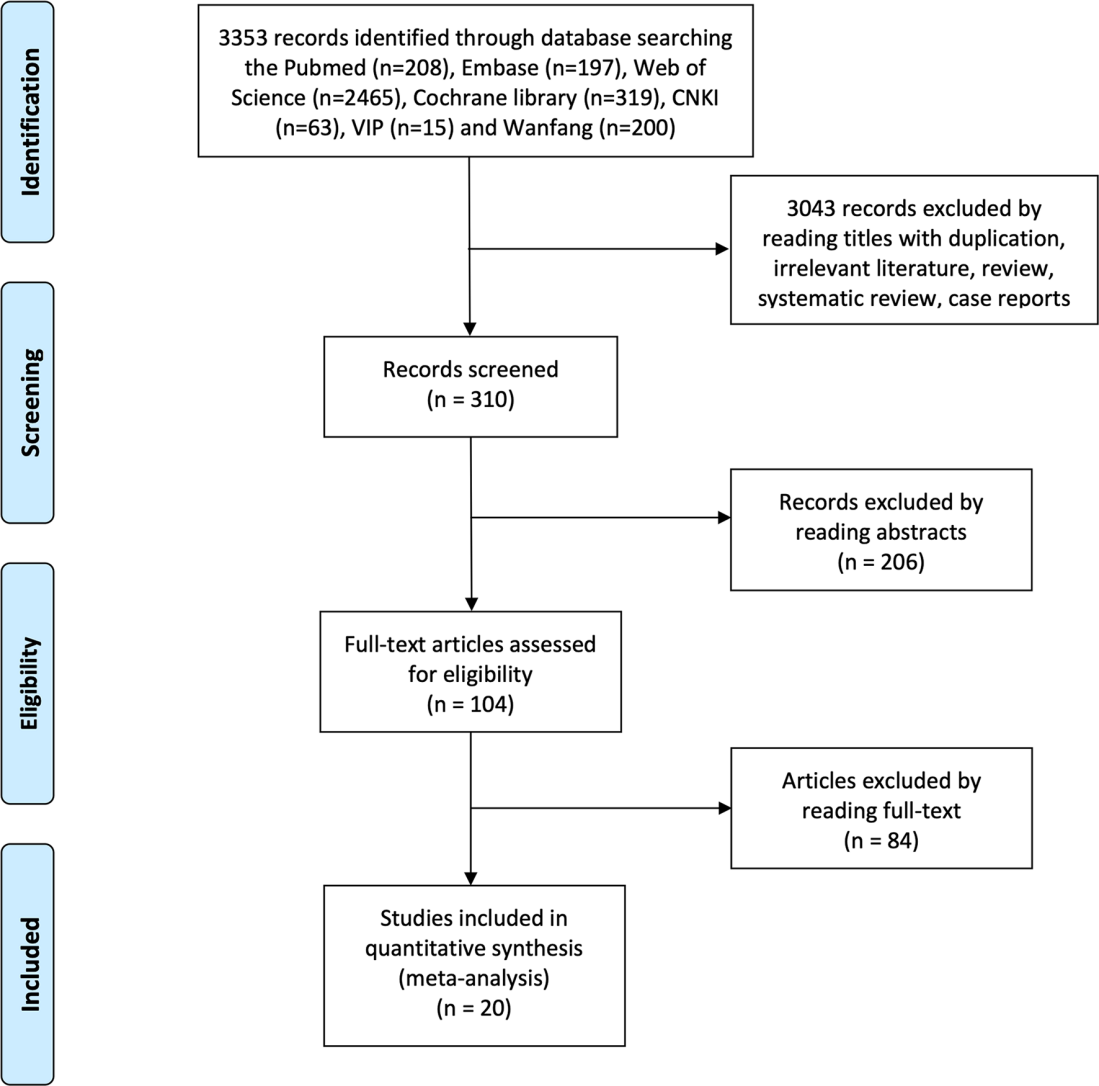
Flowchart illustrating the study selection of downstaging prior to HR.

The flowchart of the study selection process is shown in **Figure 1**. After searching seven electronic databases, 3,348 citations were retrieved initially, of which 310 for screening titles and abstracts and 66 full-text for reviewing. A total of 20 studies were finally included in the meta-analysis (**Table 1**). The quality assessment of the included studies is displayed in **Supplementary Tables 1–3**. Two prospective single-arm studies had a moderate risk of bias, and six observational studies received NOS scores of ≥7, while 12 case series had a high risk of bias. In total, most inclusive studies were rated as low-moderate quality. There were no RCT identified.

**Table 1:** Studies of downstaging therapy for hepatic resection of HCC.

Study	Year	Intervention	Types of intervention	N of receiving downstaging	Design	Reason of unresectability
**Sitzmann** (19)	1993	Combined RT and CT	LRT+systemic treatment	14	Retrospective cohort	Extrahepatic metastasis; diffuse liver tumor or major vascular invasion
**Majno** (20)	1997	TACE	LRT	49	Case series	Three or more intrahepatic tumor nodules
**Fan** (21)	1998	TACE	LRT	65	Case series	Too bulky for resection or situated centrally at the hepatic hilus
**Lau2** (22)	2001	Chemoimmunotherapy	Systemic treatment	150	Case series	Extrahepatic metastasis; diffuse liver tumor or major vascular invasion
**Clavien** (23)	2002	CT	Systemic treatment	28*	Prospective pilot study	Diffuse liver tumor; large solitary tumor or major vascular invasion
**Lau1** (24)	2004	SIR/CT	LRT/systemic treatment	71 (SIR)	Case series	Extrahepatic metastasis; diffuse liver tumor or major vascular invasion
				124 (PAIF)		
				75 (doxorubicin)		
**Tang** (25)	2004	Multimodality	LRT	379 (HAI)	Case series	NA
				1085 (HAI+HAL)		
				562 (HAI+HAL+RAIT)		
**Zhao** (26)	2009	TACE or TACE+PEI or TACE-RT	LRT	34	Case series	Too bulky for resection; diffuse liver tumor or major vascular invasion
**Shi** (27)	2012	TACE	LRT	412	Case series	Too bulky for resection or located centrally at the hepatic hilus
**Chen** (28)	2013	TACE	LRT	433	Case series	NA
**Kaseb** (29)	2013	CT (mPAIF *vs*. PAIF)	Systemic treatment	117 (33 *vs*. 84)	Retrospective cohort	Extrahepatic metastasis; diffuse liver tumor or major vascular invasion
**Lee1** (30)	2014	HAI+CCRT followed by resection *vs*. HAI+CCRT alone	LRT	243 (41 *vs*. 202)	Retrospective cohort	Too bulky for resection; diffuse liver tumor or major vascular invasion
**Lee2** (31)	2014	CCRT	LRT	41	Retrospective cohort	Too bulky for resection; major vascular invasion
**Zhang** (32)	2016	TACE-RT	LRT	82 (43 *vs*. 39)	Retrospective cohort	NA
**Li** (33)	2017	TACE+sorafinib	LRT+systemic treatment	21	Case series	BCLC stage B-C
**Hamaoka** (34)	2017	HAI+RT followed by resection *vs*. HAI+RT alone	LRT	50 (7 *vs*. 43)	Retrospective cohort	HCC with PVTT
**He** (35)	2018	HAI+sorafenib	LRT+systemic treatment	35	Prospective single-arm	Extrahepatic metastasis; HCC with PVTT
**Lee3** (36)	2019	HAI	LRT	103	Case series	Extrahepatic metastasis; diffuse liver tumor or major vascular invasion
**Goto** (37)	2020	HAI	LRT	18	Retrospective cohort	Diffuse liver tumor or major vascular invasion
**Chiu** (38)	2020	DEB-TACE *vs*. cTACE	LRT	61(42 *vs*. 19)	Retrospective cohort	Diffuse liver tumor or major vascular invasion

N, number of patients; NA, not available; RT, radiotherapy; CT, chemotherapy; TACE, transarterial chemoembolization; SIR, selective internal radiation; PAIF, cisplatin, doxorubicin, 5-fluorouracil, and interferon-alpha; HAI, hepatic arterial infusion; PEI, percutaneous ethanol injection; RAIT, radioimmunotherapy; mPAIF, modified PAIF; BCLC, Barcelona Clinic Liver Cancer; DEB-TACE, drug-eluting beads transarterial chemoembolization; cTACE, conventional transarterial chemoembolization.

*5 HCC and 23 metastatic colorectal cancer.

### Downstaging Rate

**Figure 2:**
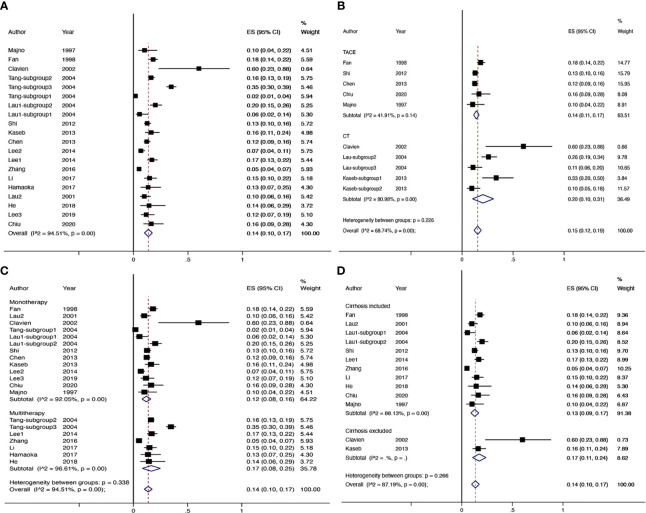
The overall downstaging success rate of hepatic resection of HCC **(A)**, pooled downstaging rate stratified by TACE and CT **(B)**, pooled downstaging rate stratified by mono/multitherapy **(C)**; pooled downstaging rate stratified by cirrhosis included/excluded **(D)**. TACE, transarterial chemoembolization; CT, chemotherapy.

There were 17 studies (4 comparative studies and 13 non-comparative studies) covering 20 subgroups with 4,878 patients that evaluated downstaging rate. There was only one case–control study comparing two chemotherapy regimens of downstaging; therefore, a single-arm meta-analysis was conducted for the downstaging success rate (**Figure 2A**). The pooled downstaging rate of HR was 14% (95% CI 0.10–0.17) with significant heterogeneity (*I* ^2^ = 94.51%). Subgroup analyses were performed by intervention, mono/multitherapy, and if patients with cirrhosis or extrahepatic spread were included. In the intervention group, data were sufficient to perform subgroup analysis on TACE and chemotherapy; the downstaging success rate displayed nonsignificant reduction in the TACE group (*I* ^2^ = 41.9%) compared to that in the chemotherapy group (*I* ^2^ = 80.98%) (20% *vs*. 14%; *p* = 0.226; **Figure 2B**), and the statistical heterogeneity decreased. Meanwhile, the downstaging rate of combination therapy (*I* ^2^ = 96.61%) demonstrated a non-significant trend towards improvement over monotherapy (*I* ^2^ = 92.05%) nonsignificantly (17% *vs*. 12%, *p* = 0.338; **Figure 2C**). Studies that included patients with cirrhosis (*I* ^2^ = 88.13%) had a lower success rate of downstaging than studies that did not, although the difference was not significant (*I* ^2^ = NA) (13% *vs*. 17%, p = 0.266; **Figure 2D**). In addition, extrahepatic spread did not exhibit a link with downstaging rate (*I* ^2^ = 83.05%) (14% *vs*. 11%, p = 0.273; **Supplementary Figure 1**).

### DFS Rate

**Figure 3:**
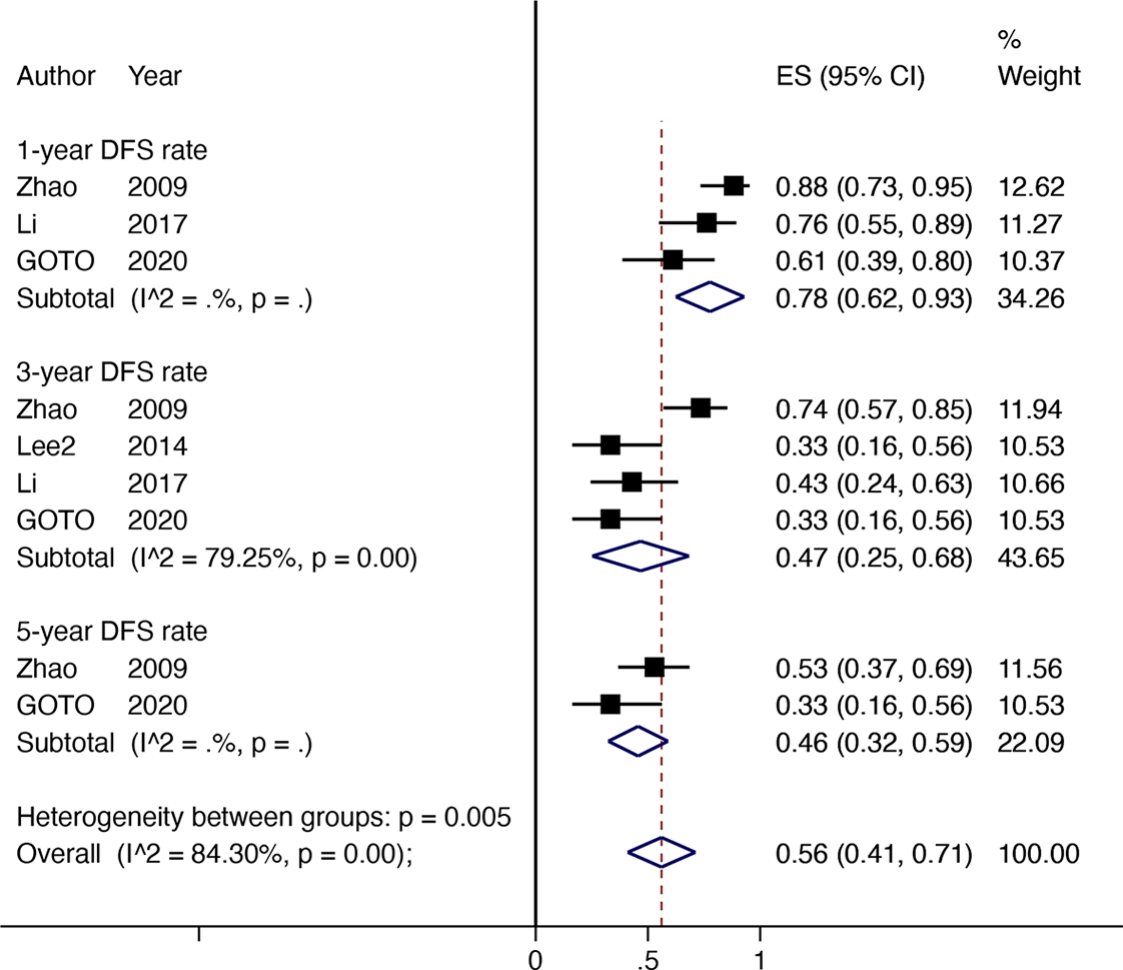
The DFS rate for HR with downstaging therapies in patients with HCC.

Four non-comparative studies with 142 patients investigated the DFS or RFS rate (referred to later in this paper as DFS). Three articles reported 1-year DFS rate; four reported 3-year DFS rates, and two reported 5-year DFS rates. The 1-year DFS rate was 78% (95% CI 0.62–0.93, *I* ^2^ = NA), 3-year DFS rate was 47% (95% CI 0.25–0.68, *I* ^2^ = 79.25%), and the 5-year DFS rate was 46% (95% CI 0.32–0.59, *I* ^2^ = NA) (**Figure 3**).

### OS Rate

**Figure 4:**
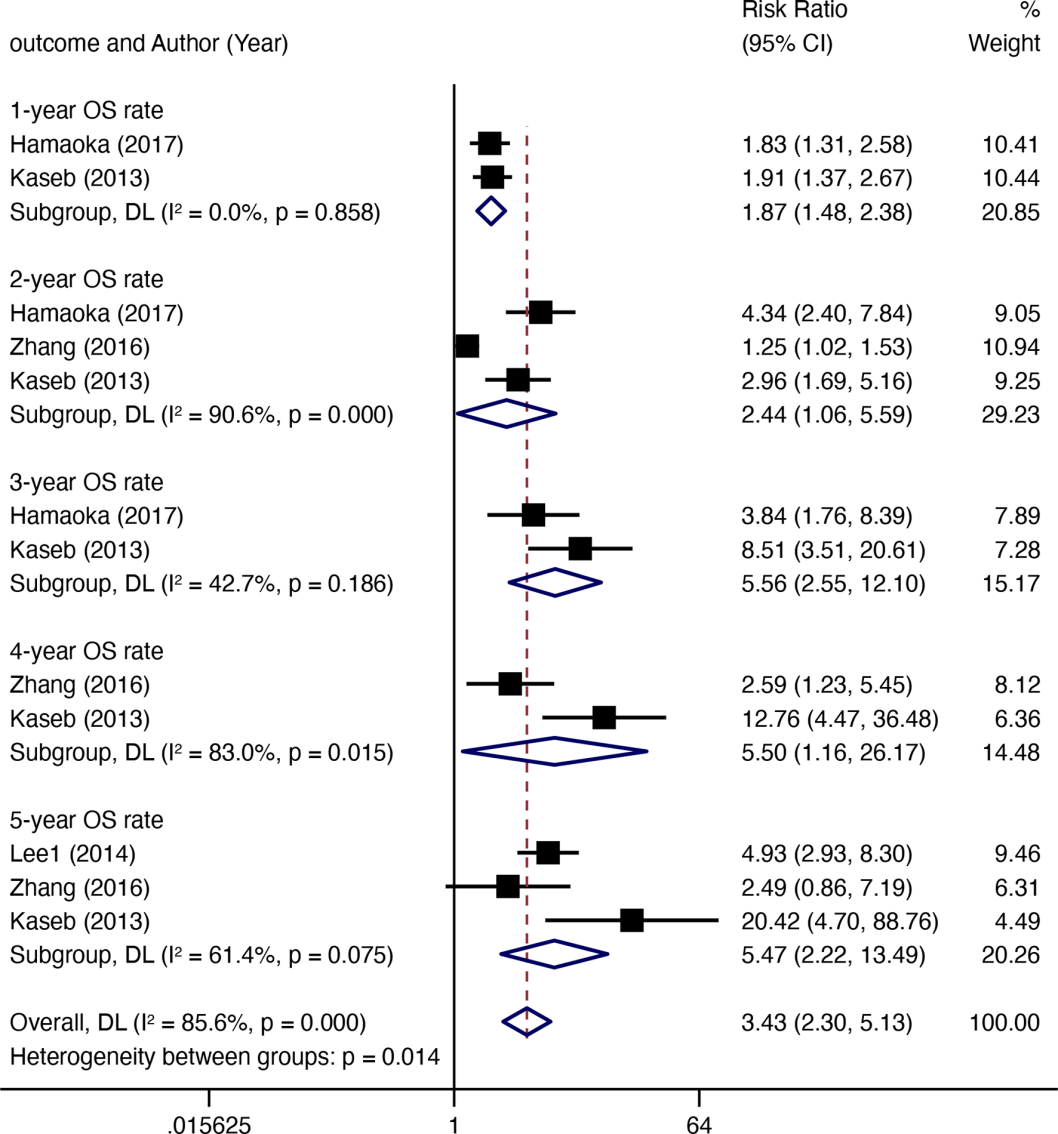
The OS rate (resection after downstaging versus LRT or systematic treatment alone) for HR with downstaging therapies in patients with HCC.

**Figure 5:**
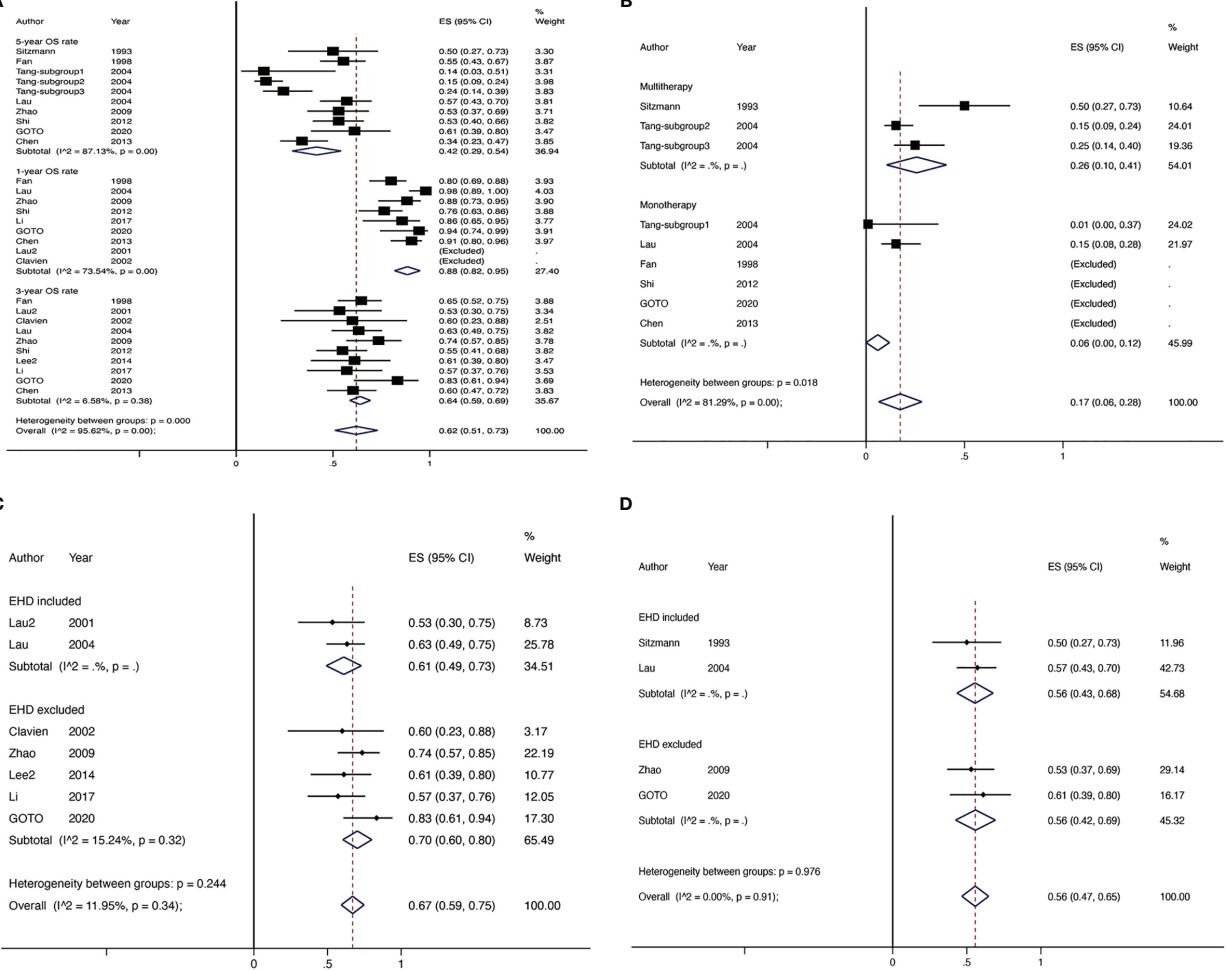
The OS rate of non-comparative studies for HR with downstaging therapies in patients with HCC **(A)**, the 5-year OS rate of non-comparative studies for HR with downstaging therapies stratified by mono/multitherapy **(B)**, the 3-year OS rate of non-comparative studies for HR with downstaging therapies stratified by EHD included/excluded **(C)**, the 5-year OS rate of non-comparative studies for HR with downstaging therapies stratified by EHD included/excluded **(D)**. EHD, extrahepatic disease.

Four comparative studies enrolled 492 patients documented data for OS rates (**Figure 4**). Compared with non-surgical treatment, HR after downstaging therapy showed a significant increase in the 1-year OS rate (RR 1.87, 95% CI 1.48–2.38, *I* ^2^ = 0.0%), 2-year OS rate (RR 2.44, 95% CI 1.06–5.59, *I* ^2^ = 90.6%), 3-year OS rate (RR 5.56, 95CI 2.55–12.10, *I* ^2^ = 42.7%), 4-year OS rate (RR 5.56, 95% CI 2.55–12.10, *I* ^2^ = 42.7%), and 5-year OS rate (RR 5.47, 95% CI 2.22–13.49, *I* ^2^ = 61.4%). Twelve non-comparative studies investigated the OS rate in 618 patients in 17 subgroups (**Figure 5A**). Eight articles reported 1-year OS rates, 10 reported 3-year OS rates, and 9 reported 5-year OS rates for 10 subgroups. The 1-year pooled OS rate (*I* ^2^ = 73.54%) was 88% (95% CI 0.82–0.95), the 3-year pooled OS rate (*I* ^2 =^ 6.58%) was 64% (95% CI 0.59–0.69), and the 5-year combined OS rate (*I* ^2 =^ 87.13%) was 42% (95% CI 0.29–0.54). Subgroup analysis showed that the 3-year OS rate of extrahepatic disease was higher than that of the subgroup without extrahepatic disease, but the difference was not statistically significant. The 5-year OS rate in patients with extrahepatic disease was comparable to that in patients without extrahepatic disease (**Figures 5C, D**). Subgroup analysis by modality for the 5-year OS rate revealed that the efficacy of multitherapy was better than that of monotherapy (0.26 *vs*. 0.06, *p* = 0.02) (**Figure 5B**).

## Discussion

Unresectable HCC is generally considered incurable. However, the definition of resectable/unresectable is subjective in accordance with the extent of tumor, functional liver reserve, and surgeons’ judgments. Although conversion surgery may be applied following adequate downstaging achieved by tumor downsizing methods or increasing the future liver remnant (FLR), a common criticism is that technically resectable does not represent the optimal oncological outcome. Thus, we conducted this meta-analysis to determine whether downstaging therapies aimed at shrinking tumors are feasible or effective for unresectable intermediate and advanced HCC.

Our meta-analysis is the first to synthesize the existing evidence on the success rates and effectiveness of LRT and/or systemic treatment as downstaging strategies prior to HR in patients with advanced unresectable HCC, and it confirms that only approximately 14% of patients attain downstaging after initiation of HR. The chemotherapy, combination, and non-cirrhosis groups exhibited higher rates of downstaging, but these differences were not significant. The conversion rate of HR is much lower than that of LT (39), which remains unsatisfactory. This might be explained by the fact that studies on downstaging for HR were fewer in number and of poorer quality compared to studies of LT, which often included LRT as a bridge to transplantation, and some studies enrolled patients who failed previous treatment. There is significant heterogeneity in terms of different inclusion criteria of downstaging, subjective judgment of resectability, and the selection of LRT, which is closely related to the experiences and preferences of each institution. In comparative studies, the OS rates of resection after downstaging were notably better than those in patients receiving LRT or systemic treatment alone at 1 year (RR 1.87, 95% CI 1.48–2.38), 3 years (RR 5.56, 95% CI 2.55–12.10), and 5 years (RR 5.47, 95% CI 2.22–13.49). In comparative studies, the pooled DFS rates of patients undergoing HR after successful downstaging were 78% (95% CI 0.62–0.93) at 1 year, 47% (95% CI 0.25–0.68) at 3 years, and 46% (95% CI 0.32–0.59) at 5 years. The pooled OS rates were 88% (95% CI 0.82–0.95) at 1 year, 64% (95% CI 0.59–0.69) at 3 years, and 42% (95% CI 0.29–0.54) at 5 years. These results were better than the reported data of non-surgical interventions (40), and worse than the industry-accepted survival rates of early HCC (4). As a result, downstaging may be considered an alternative strategy for patients with unresectable HCC. In this paper, downstaging is defined as systemic therapy or regional antitumor therapy with the aim of converting unresectable HCC into resectable HCC. It is necessary to optimize the downstaging strategies to further improve the effect from an intent-to-treat viewpoint. Although many studies have investigated the clinical efficacy of LRT or systemic therapy alone for unresectable HCC, the best intervention for downstaging therapy is not yet clear. The causes of unresectability varied in different studies, mainly including local extension, major vascular invasion, and extrahepatic spread. LRTs such as TACE, SIR, and HAI tend to be adopted in patients with local extension and major vascular invasion. TACE is considered a standard care for unresectable HCC and has also been widely used in downstaging strategies. Recently, TARE/SIR, as an alternative to TACE, has received increasing attention because it can effectively shrink a tumor and shorten the response time, and may be adopted for the patients of portal thrombosis (a contraindication of TACE) (41). Microwave and radiofrequency ablation have been used in LT downstaging, but there are no related reports on HR downstaging. Six articles reported patients with extrahepatic metastases, mainly using systemic treatment. LRT was only employed in one study, which showed that extrahepatic metastasis was not a significant factor in survival for patients with HAI with chemotherapy (36). The use of HAI with chemotherapy in HCC patients with minimal extrahepatic metastasis was supported in previous studies, for intrahepatic lesions have a greater impact on survival than extrahepatic lesions (42–44). Based on our meta-analysis, extrahepatic metastasis was not associated with the 3- and 5-year OS rate. Furthermore, the subgroup analyses of the downstaging rate on intervention and patients with or without cirrhosis showed no significant difference in the downstaging rate, while these data were insufficient for subgroup analysis on survival. Notably, the subgroup analysis of mono/multitherapy showed that multimodality downstaging prolongs long-term survival. This suggests that the results may not be affected by the selection of downstaging therapy, but more influenced by whether the combination of downstaging therapy is implemented.

Another important issue is the endpoint of downstaging and criteria for post-downstaging liver resection, which must be defined more precisely. It is generally believed that a reduction in tumor size may be an effective evaluation indication (20, 21, 34). A few researchers have adopted the criteria of partial remission (PR) for resection after downstaging (21, 27). However, whether the subsequent HR needs to be removed when the tumor disappears on imaging remains controversial. Shi et al. (2012) considered that surgical resection should be performed under these conditions because clinical complete response (CR) does not represent pathological CR (27). Residual viable cancer cells may lead to a high recurrence rate (10). According to Hamaoka et al. (2017) (34) and Lee et al. (2019) (36), successful conversion to surgery is considered to be a factor of favorable prognosis. In contrast, Zhang et al. (2016) reported that salvage surgery after TACE for unresectable HCC has an OS benefit only in patients with a PR to TACE, while those achieving CR group do not show improvement (32). This might be due to the role of downstaging in the selection of biological aggression. The presence of vascular invasion, multiple tumors, and high alpha-fetoprotein levels are regarded as risk factors of survival in patients with resectable HCC as well as downstaging (2, 21, 31, 35). Therefore, downstaging may serve as a screening tool to identify patients who might benefit from surgery. Since conventional criteria for HR are based on HCC morphology, downstaging, which can predict HCC biology, will be more favorable.

The limitation of high heterogeneity among different downstaging strategies for HCC should also be considered. The selection of a treatment strategy is based merely on the habit at a single center. Additionally, no RCT has been performed; therefore, the grade of evidence was weak.

To date, there has been surrounding the best strategy for unresectable HCC. The evidence of HR mainly came from small published series, having demonstrated useful attempts. Based on this meta-analysis, operable patients with unresectable HCC may be screened for downstaging. Surgical resection after successful downstaging can maximize the improvements in the prognosis of patients with unresectable HCC, bringing hope for patients initially considered incurable. With the rapid advancements in LRT in recent years, the emergence of novel targeted therapies, especially immunotherapy, has tremendously facilitated non-surgical treatments for HCC, suggesting a potential role for downstaging. In the future, prospective trials with large sample sizes on these new methods are expected to provide reasonable guidance and inspire more effective strategies for downstaging approaches. Future criteria should include a clear downstaging endpoint and molecular biological information and markers.

## Data Availability Statement

The raw data supporting the conclusions of this article will be made available by the authors, without undue reservation.

## Ethics Statement

Ethical review and approval was not required for the study on human participants in accordance with the local legislation and institutional requirements. Written informed consent for participation was not required for this study in accordance with the national legislation and the institutional requirements.

## Author Contributions

Conceptualization: LeL and JY. Study selection and data extraction: XC and LiL. Statistical analysis: XC. Writing—original draft: XC and LiL. Writing—review and editing: LeL and JY. Final approval of the manuscript: all authors. All authors contributed to the article and approved the submitted version.

## Funding

This research was supported by the Basic Ability Enhancement Program for Young and Middle-aged Teachers in Higher Education Institutions of Guangxi (Nos. 2021KY0091 and 2021KY0283) and the Science Foundation for Distinguished Young Scholars of Guangxi University of Chinese Medicine (Grant No. 2020JQ001).

## Conflict of Interest

The authors declare that the research was conducted in the absence of any commercial or financial relationships that could be construed as a potential conflict of interest.

## Publisher’s Note

All claims expressed in this article are solely those of the authors and do not necessarily represent those of their affiliated organizations, or those of the publisher, the editors and the reviewers. Any product that may be evaluated in this article, or claim that may be made by its manufacturer, is not guaranteed or endorsed by the publisher.
