# Supplementary material for: Downstaging Therapies for Unresectable Hepatocellular Carcinoma Prior to Hepatic Resection: A Systematic Review and Meta-Analysis
Source: Front Oncol. 2021 Nov 19;11:740762. doi: 10.3389/fonc.2021.740762 (PMC8639517; doi:10.3389/fonc.2021.740762)
Supplement: Supplementary file 1 [file DataSheet_1.docx]

**Supplementary Table 1 Risk of bias assessment for single-arm studies included in the meta-analysis using the MINORS tool**

| Study | A clearly stated aim | Inclusion of consecutive patients | Prospective data collection | End points appropriate to the aim of the study | Unbiased assessment of the study end point | Follow-up period appropriate to the aim of the study | Loss of follow-up lower than 5% | Prospective calculation of the study size | Total score | Risk of bias |
| --- | --- | --- | --- | --- | --- | --- | --- | --- | --- | --- |
| He 2018 | 2 | 1 | 2 | 2 | 0 | 1 | 2 | 0 | 10 | Medium |
| Clavien 2001 | 2 | 2 | 1 | 2 | 1 | 1 | 2 | 0 | 11 | Medium |

**Supplemental Table 2 Risk of bias assessment for case series included in the meta-analysis using the IHEQA tool**

| Criteria | Lee3 2019 | Lau2 2001 | Lau1 2004 | Li 2017 | Lee2 2014 | Chen 2013 | Zhao 2019 | Tang 2004 | Sitzmann1993 | Fan 1998 | Majno1997 | Shi 2012 |
| --- | --- | --- | --- | --- | --- | --- | --- | --- | --- | --- | --- | --- |
| **﻿Study objective** |  |  |  |  |  |  |  |  |  |  |  |  |
| ﻿1. Is the hypothesis/aim/objective stated clearly in the abstract, introduction or methods section? | Y | Y | Y | Y | Y | Y | Y | Y | Y | Y | Y | Y |
| **﻿Study population** |  |  |  |  |  |  |  |  |  |  |  |  |
| ﻿2. Are the characteristics of the participants included in the study described? | Y | Y | Y | Y | Y | Y | Y | Y | Y | Y | Y | Y |
| ﻿3. Were the cases collected in more than one centre? | N | N | N | N | U | U | Y | N | U | N | N | U |
| ﻿4. Are the eligibility criteria (inclusion and exclusion criteria) for entry into the study explicit and appropriate? | Y | N | Y | Y | Y | N | P | Y | Y | Y | Y | P |
| ﻿5. Were participants recruited consecutively? | U | Y | Y | U | U | U | U | U | Y | U | U | N |
| ﻿6. Did participants enter the study at a similar point in the disease? | Y | Y | Y | Y | Y | Y | Y | Y | Y | Y | Y | Y |
| **﻿Intervention and co-intervention** |  |  |  |  |  |  |  |  |  |  |  |  |
| ﻿7. Was the intervention clearly described in the study? | Y | Y | Y | Y | Y | Y | Y | Y | Y | Y | Y | Y |
| ﻿8. Were additional interventions (co-interventions) clearly reported in the study? | Y | Y | Y | Y | Y | Y | Y | Y | Y | Y | Y | Y |
| **﻿Outcome measure** |  |  |  |  |  |  |  |  |  |  |  |  |
| ﻿9. Are the outcome measures clearly defined in the introduction or methods section? | Y | Y | Y | N | Y | Y | Y | Y | Y | N | N | Y |
| ﻿10. Were relevant outcomes appropriately measured with objective and/or subjective methods? | Y | U | Y | Y | Y | U | U | Y | Y | Y | Y | Y |
| ﻿11. Were outcomes measured before and after intervention? | Y | Y | N | N | Y | N | N | N | N | N | N | N |
| **﻿Statistical analysis** |  |  |  |  |  |  |  |  |  |  |  |  |
| ﻿12. Were the statistical tests used to assess the relevant outcomes appropriate? | U | U | U | U | U | U | U | U | Y | U | Y | N |
| **﻿Results and conclusions** |  |  |  |  |  |  |  |  |  |  |  |  |
| ﻿13. Was the length of follow-up reported? | N | Y | Y | Y | N | Y | Y | Y | N | N | Y | Y |
| ﻿14. Was the loss to follow-up reported? | N | N | N | N | N | Y | N | N | N | N | N | N |
| ﻿15. Does the study provide estimates of the random variability in the data? | Y | N | Y | N | N | Y | N | N | N | N | Y | N |
| **﻿Analysis of relevant outcomes?** |  |  |  |  |  |  |  |  |  |  |  |  |
| ﻿16. Are adverse events reported? | N | Y | Y | Y | N | N | Y | N | Y | Y | Y | Y |
| ﻿17. Are the conclusions of the study supported by results? | Y | Y | Y | Y | Y | Y | Y | Y | Y | Y | Y | Y |
| **﻿Competing interests and sources of support** |  |  |  |  |  |  |  |  |  |  |  |  |
| ﻿18. Are both competing interests and sources of support for the study reported? | Y | N | N | N | N | N | N | Y | N | N | N | N |
| **New criteria** |  |  |  |  |  |  |  |  |  |  |  |  |
| 19. ﻿Was the study conducted prospectively? | N | N | N | N | N | N | N | N | N | N | N | N |
| 20. ﻿Were the main outcomes assessed blind to/independent of intervention status? | U | U | U | U | U | U | U | U | U | U | U | U |

Y, yes; N, no; N/A, not applicable; U, unclear from study report; P, partially resported.

**Supplemental Table 3 Risk of bias assessment for cohorts included in the meta-analysis using the NOS tool**

| **Study** | **Selection** | | | | |  | **Comparability** |  | **Outcome** | | | | **NOS scores** |
| --- | --- | --- | --- | --- | --- | --- | --- | --- | --- | --- | --- | --- | --- |
|  | **Representativeness of the exposed cohort** | | **Selection of the non-exposed cohort** | **Ascertainment**  **of exposure** | **Demonstration that outcome of interest was not present at start of study** |  | **Comparability of cohort based on the design or analysis** |  | | **Assessment**  **of outcome** | **Was follow-up long enough for outcome to occur** | **Adequate of**  **follow up of**  **cohort** |  |
| Kaseb 2013 | | 1 | 1 | 1 | 0 |  | 1 |  | | 1 | 1 | 1 | 7 |
| Lee1 2014 | | 1 | 1 | 1 | 0 |  | 1 |  | | 1 | 1 | 1 | 7 |
| Hamaoka 2017 | | 1 | 1 | 1 | 0 |  | 1 |  | | 1 | 1 | 1 | 7 |
| Zhang 2016 | | 1 | 1 | 1 | 1 |  | 1 |  | | 1 | 1 | 1 | 8 |
| GOTO 2020 | | 1 | 1 | 1 | 0 |  | 1 |  | | 1 | 1 | 1 | 7 |
| Chiu2020 | | 1 | 1 | 1 | 1 |  | 1 |  | | 1 | 0 | 1 | 7 |


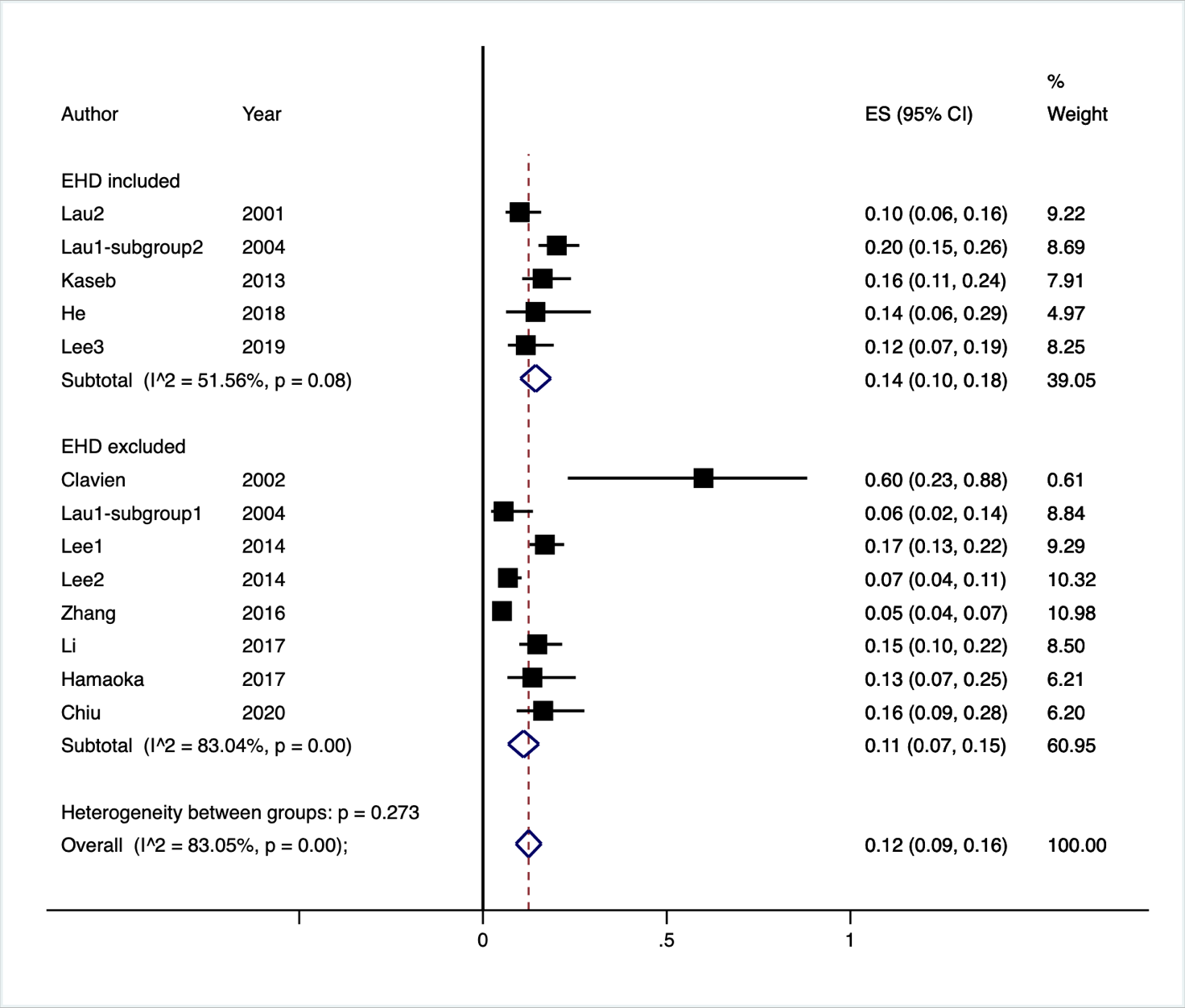


**Abbreviations:** EHD, extrahepatic disease

**Supplementary Figure 1 The pooled downstaging rate stratified by EHD**
